# Exploring the link between glycated serum protein and esophageal metrics in diabetic GERD patients

**DOI:** 10.1515/med-2025-1373

**Published:** 2026-03-09

**Authors:** JingXia Wang, QiaoQiao Kong, Jing Chen, HePing Xia, MaoLin Han, ChuangZhou Xie, Ning You

**Affiliations:** Ophthalmology, Stomatology and Otorhinolaryngology – Head and Neck Surgery, Head and Neck Surgery, The Affiliated Hospital of Bei-Hua University, Jilin, Jilin Province, China; Department of Critical Care Medicine, Yantai Qishan Hospital, Yantai, Shandong Province, China; Department of Neurology, Xingyuan Hospital of Yulin (4TH Hosptial of Yulin), Yulin, Shaanxi Province, China; Department of Internal Medicine, People’s Hospital of Dingbian County, Yulin, Shaanxi Province, China; Department of General Surgery, People’s Hospital of Dingbian County, Yulin, Shaanxi Province, China; Department of Vascular Surgery, Sengong Hospital of Shaanxi Province, Xi’an, Shaanxi Province, China; Department of Gastroenterology, People’s Hospital of Fugu County, Yulin City, Shaanxi Province, China

**Keywords:** diabetes mellitus, gastroesophageal reflux disease, glycated serum protein, post-reflux swallow-induced peristaltic wave, esophageal baseline impedance

## Abstract

**Objectives:**

This study assessed the relationship between glycated serum protein (GSP) and post-reflux swallow-induced peristaltic wave (PSPW) index and esophageal baseline impedance (BI) in patients with diabetes mellitus (DM) with gastroesophageal reflux disease (GERD).

**Methods:**

General clinical data of patients in the DM and DM/GERD groups were compared. Multifactorial Logistic regression analyses were performed to screen for independent factors on GERD in DM patients, and odds ratio (OR) was calculated. The risk of GERD was assessed using a spline regression model constructed on the basis of GSP, PSPW and BI. Predictive efficacy was analyzed using Receiver operating curve (ROC) curve analysis.

**Results:**

Statistically significant differences were found in TG, hs-CRP, GSP, PSPW, and BI between patients in the DM and DM/GERD groups. PSPW and BI (OR=0.99, p<0.01) were independent protective factors, and elevated GSP was an independent risk factor for the development of GERD. A linear relationship was validated between GSP and PSPW and BI and the prevalence risk of developing GERD in patients with DM, all with good predictive efficacy.

**Conclusions:**

GSP, PSPW, and BI show significant correlations with the onset and progression of GERD in patients and all demonstrate good predictive efficacy. However, due to the small size of the patient cohort, the generalizability of these findings requires further validation.

## Introduction

Diabetes mellitus (DM) is a metabolic disorder characterized by hyperglycemia due to insulin resistance or insulin secretion defects [1]. Patients with DM develop a variety of complications, and these patients frequently present with gastrointestinal symptoms. In gastroesophageal reflux disease (GERD), gastric contents reflux into the esophagus, resulting in severe symptoms and complications [2]. Patients with GERD usually complain of heartburn and acid reflux, but some suffer atypical symptoms such as burning throat, hoarseness, chest pain, watery rash, chronic cough, halitosis, and dysphagia [3]. DM is often identified as a risk factor for GERD [4]. There has been conflicting evidence regarding the relationship between DM and GERD. Some studies have found a positive correlation [5], [6], [7], whereas others have found no association [8], 9].

Glycated serum protein (GSP), which consists of 70 % ALB, is formed by a non-enzymatic oxidation that occurs when blood glucose binds to plasma proteins. Since serum proteins are synthesized faster than haemoglobin [10], the concentration of GSP reflects the blood glucose over the last 1–3 weeks and is not affected by temporary fluctuations in blood glucose [11]. Glycated hemoglobin reflects the average blood glucose concentration over the past 9–12 weeks, and GSP is better in reflecting the effectiveness of glycemic control, and is more sensitive and timely in reflecting blood glucose levels. In clinical practice, serum albumin can also reflect the average blood glucose in DM patients in the 2–3 weeks prior to the measurement, but GA is more affected by body mass index and thyroid hormones, etc. [12]. Based on this, the present study was conducted to assess blood glucose in DM patients by detecting GSP.

In GERD, refluxing gastric contents cause the distended esophageal wall to activate tensor receptors and induces secondary peristalsis, forcing the refluxed mass to return rapidly to the stomach. After an episode of reflux, peristaltic waves triggered by the esophageal stretch reflex initially clear the volume of reflux. Subsequently, chemical clearance occurs via the vagus nerve-mediated esophageal-salivary reflex. Acidification of the distal esophagus leads to a threefold increase in salivary secretion, which induces a peristaltic wave. PSPW, a peristaltic defence mechanism, involves paracolic esophageal contractions in response to reflux, reflecting peristalsis’ ability to remove reflux from the esophagus. MII-pH monitoring is more accurate when using the PSPW index. Erosive reflux disease (ERD) and non-ERD patients show lower PSPW index than functional heartburn patients [13]. The BI represents the state of mucosal integrity [14]. In acid-perfused esophageal mucosa, BI measures are used to assess mucosal integrity and are correlated with trans-epithelial resistance (TER) [15]. Dilated intercellular space (DIS) is often considered a structural marker of GERD as it reflects early impairments in the esophageal mucosa [16]. The occurrence of DIS has been associated with a reduction in TER [17].

GSP, PSPW, and BI may be closely associated with the occurrence of GERD in DM patients. The aim of this study was to assess the predictive efficacy of GSP, PSPW and BI on the occurrence of GERD.

## Materials and methods

### Study population

DM patients with persistent typical GERD symptoms were collected from January 2022 to December 2023 according to the Montreal consensus for MII-pH monitoring.

Inclusion criteria: patients aged ≥18 years; patients who met the diagnostic criteria of the Los Angeles classification and the diagnostic DM criteria established by the American Diabetes Association in 2020; patients with stable conditions who could cooperate in completing the questionnaire; patients who underwent gastric endoscopy 1 week or less prior to the MII-pH monitoring; patients with uncomplicated malignant tumors; patients with no other immune system diseases; patients with complete clinical data and follow-up data. The present study was approved by the Ethics Committee of The Affiliated Hospital of Bei-Hua University (No: 2021BH746; Date: 2021.06.10) and written informed consent was provided by all patients prior to the study start. All procedures were performed in accordance with the ethical standards of the Institutional Review Board and The Declaration of Helsinki, and its later amendments or comparable ethical standards.

Exclusion criteria: patients taking medications that may affect esophageal motor function or gastric acid secretion; patients with a history of gastrointestinal tumor, peptic ulcer, or surgery; patients with primary or secondary severe esophageal dyskinesia; patients with co-morbidities of severe infectious diseases; patients with co-morbidities of other hematological disorders; patients with severe cardiac disease or renal dysfunctions; patients with abnormalities other than chronic superficial gastritis or erosive esophagitis; patients with missing clinical data; patients with symptom frequency less than weekly.

### Diagnostic criteria

All patients completed the GERDQ questionnaire. The GERDQ questionnaire consists of 6 questions, showing a sensitivity of 65 % and a specificity of 71 % for the diagnosis of GERD. Subjects who scored higher than 8 are diagnosed as abnormal [18]. All patients underwent gastric endoscopy and MII-pH monitoring. All study procedures were performed by five endoscopists. The Los Angeles classification was used as a diagnostic criterion for reflux esophagitis. GERD was diagnosed when patients had erosive esophagitis or non-erosive esophagitis with a questionnaire score of ≥8 with minimal change. If there was no visible esophageal mucosal injury during endoscopy and if there was a probability of esophageal acid exposure time (>4.2 %) and/or symptom association probability (>95 %) with acid/non-acid reflux during MII-pH monitoring, MII-pH monitoring and gastric endoscopy identified healthy asymptomatic controls.

### General information

General information about the patients was collected, including age, body mass index, and routine preoperative examination. The duration of diabetes and history of diabetes mellitus were also assessed.

### Laboratory indicators

Serum specimens were collected from all patients by collecting 3–4 mL of fasting venous blood in the early morning and centrifuging at 3,500 r/min for 8 min. Blood biochemical parameters were measured by Beckmann 780, including fasting blood glucose, lipid levels, aspartate transaminase, alanine aminotransferase, Blood Urea Nitrogen, γ-lutamyltranspeptidase, and creatinine. Hypersensitive C-reactive protein (hs-CRP) was detected by biochemical analyzer (Roche, model Cobas C501) and immunoturbidimetric assay (Beijing Bangding Biomedicine Company). Lipid metabolism indices, i.e., high-density lipoprotein cholesterol (HDL), low-density lipoprotein cholesterol (LDL), total cholesterol (TC), and triacylglycerol (TG) levels, were measured using a Hitachi 7060 automated biochemistry analyzer.

### MII-pH indicators

BioView analysis software (Sandhill Scientific Inc.) was used to analyze BI and pH values of reflux events. An operator who was unaware of all clinical details manually analyzed BI-pH curves within a 2-min timeframe. It was defined as a reflux event when there was a drop in impedance by 50 % over the pre-swallow baseline, followed by a return to the pre-swallow baseline, with retrograde flow from the distal impedance channel. The most proximal channel of impedance decline determined whether the reflux event occurred in the pharynx, proximal esophagus or distal esophagus. Depending on the pH of the reflux, it was classified as acidic (pH<4) or non-acidic (pH 24). PSPW is characterized as a swallowing action identified by the highest esophageal impedance channel and maintained up to the distal channel within 30 s post-reflux event, excluding PSPW that didn’t start near the end or fail to arrive at the distal impedance electrode. The PSPW index was calculated by dividing the number of reflux with PSPW within 30 s by the total number of reflux events. Esophageal BI was calculated by BioView Analysis, BL_Plot program V2.0 (Sandhill Scientific).

### Statistical analyses

SPSS 26.0 was used for statistical analysis. Measures were tested for normality, and those that met the normal distribution were expressed as mean ± standard deviation (SD), and Student’s *t*-test was used for comparison between two groups. Non-normal distribution was expressed as median and interquartile spacing M (Q25∼Q75), and comparisons between groups were made using Wilcoxon signed rank sum test. The chi-square test was performed for count data. Independent variables with statistically significant differences in univariate analysis were included in multifactorial Logistic regression analyses to screen for independent factors. A spline regression model was constructed to test whether there was a nonlinear relationship between GSP, PSPW and BI and GERD risk. The predicted efficacy was analyzed by Receiver operating curve (ROC) curve analysis. p<0.05 was considered significant difference.

### Ethics approval

The present study was approved by the Ethics Committee of The Affiliated Hospital of Bei-Hua University and written informed consent was provided by all patients prior to the study start. All procedures were performed in accordance with the ethical standards of the Institutional Review Board and The Declaration of Helsinki, and its later amendments or comparable ethical standards.

### Informed consent

Written informed consent was obtained from each subject.

## Results

### Comparison of clinical general data between patients in DM and DM/GERD groups

All enrolled patients were divided into DM group (n=124) and DM/GERD group (n=54) after GERDQ questionnaire, gastric endoscopy, and MII-pH monitoring, and the probability of GERD among DM patients was 30.33 %. Table 1 summarizes the clinicopathological characteristics of all subjects. The two groups did not differ significantly in age (p>0.05). It ranged from 55 to 61 years old for the DM group, with a mean age of 58.33 ± 3.27 years. The mean age for the DM/GERD group was 59.39 + 5.82 years; the range was 53–65 years. Apart from this, no significant differences were found in BMI, past medical history, duration of DM, and routine blood tests (AST, ALT, FBG, etc.) between the two groups (p>0.05). Notably, both TG and hs-CRP levels were significantly higher in the DM/GERD group than in the DM group (p<0.05).

**Table 1:** Comparison of clinical general data between DM group and DM/GERD group.

Characteristics	DM group (n=124)	DM/GERD group (n=54)	p-Value
Sex (male)	40 (32.26 %)	16 (29.63 %)	0.73
Age (year)	58.33 ± 3.26	59.39 ± 5.83	0.21
BMI, kg/m^2^	24.40 ± 0.37	24.31 ± 0.41	0.17
Smoking history	57 (45.97 %)	24 (44.44 %)	0.85
Drinking history	22 (17.74 %)	12 (22.22 %)	0.49
Coronary heart disease	21 (16.94 %)	8 (14.84 %)	0.73
Course of DM	8.67 ± 0.54	8.78 ± 0.86	0.39
AST, U/L	28.02 (26.47, 29.44)	28.43 (27.55, 29.14)	0.20
ALT, U/L	28.89 ± 2.36	29.40 ± 1.34	0.07
FBG, mg/dL	168.66 ± 41.3	180.01 ± 32.9	0.08
FINS, mIU/L	5.83 ± 1.54	6.11 ± 1.97	0.36
HDL-C, mg/dL	61.25 ± 15.81	58.75 ± 17.66	0.35
LDL-C, mg/dL	136.33 ± 26.50	128.55 ± 34.52	0.14
TC, mg/dL	163.00 ± 42.82	174.55 ± 33.37	0.08
TG, mg/dL	189.24 (141.73, 224.30)	200.51 (171.12, 231.93)	0.04
HbA1c, %	8.23 ± 1.58	8.6 ± 1.41	0.14
BUN, mg/dL	14.03 ± 4.79	15.02 ± 4.52	0.20
Cre, mg/dL	0.99 ± 0.17	1.03 ± 0.16	0.15
γ-GTP, U/L	31.41 (26.59, 35.80)	31.86 (25.44, 37.72)	0.68
hs-CRP, mg/L	13.04 ± 3.01	14.23 ± 3.14	0.02

Continuous data following a normal distribution were expressed as mean ± SD, and compared between groups using the independent samples *t*-test. Continuous data with a skewed distribution were expressed as median (interquartile range) and compared using the independent samples rank-sum test. Categorical data were expressed as n (%) and analyzed using the chi-square test. p<0.05 was considered statistically significant. DM, diabetes mellitus; GERD, gastroesophageal reflux disease; BMI, body mass index; AST, aspartate transaminase; ALT, alanine aminotransferase; FBG, fasting blood glucose; FINS, fasting insulin; HDL, high density lipoprotein; LDL, low density lipoprotein; TC, total cholesterol; TG, triglyceride; HbA1c, glycated hemoglobin; BUN, blood urea nitrogen; Cre, creatinine; γ-GTP, gamma-glutamyltranspeptidase; hs-CRP, hypersensitive C-reactive protein.

### GSP, PSPW and BI between patients in DM and DM/GERD groups

GSP in the DM/GERD group was higher than that in the DM group, while PSPW and BI were lower than those in the DM group (p<0.05) (Table 2).

**Table 2:** GSP, PSPW and BI between the two groups.

Characteristics	DM group (n=124)	DM/GERD group (n=54)	p-Value
GSP, μmol/L	165.99 (141.89, 202.31)	198.22 (157.56, 242.65)	<0.01
PSPW index, %	55.34 (43.43, 66.86)	43.30 (36.54, 51.42)	<0.01
BI, Ω	3,527.76 (2,811.67, 4,280.49)	2,709.60 (1930.93, 3,257.70)	<0.01

Continuous data following a normal distribution were expressed as mean ± SD, and comparisons between two groups were performed using the independent samples *t*-test. Continuous data with a skewed distribution were expressed as median (interquartile range), and comparisons between two groups were performed using the independent samples rank-sum test. p<0.05 was considered statistically significant. DM, diabetes mellitus; GERD, gastroesophageal reflux disease; GSP, glycated serum protein; PSPW, post-reflux swallow-induced peristaltic wave; BI, baseline impedance.

### Multifactorial logistic regression analysis of the occurrence of GERD in DM patients

A multifactorial logistic regression model was established (Table 3) with the factors with differences analyzed in Tables 1 and 2 as independent variables, and TG, hs-CRP, GSP, PSPW and BI as dependent variables. After adjusting for confounding factors including age, BMI, smoking and alcohol consumption, and DM duration, neither TG (OR=1.01, p=0.09) nor hs-CRP (OR=1.09, p=0.24) were correlated with the occurrence of GERD. PSPW (OR=0.93, p<0.01) and BI (OR=0.99, p<0.01) were independent protective factors, and elevated GSP (OR=1.01, p=0.01) was an independent risk factor for GERD development (p<0.05) (Table 3).

**Table 3:** Multivariate logistic regression model analysis of the risk factors of GERD in DM patients.

Indices	OR	95 % CI	p-Value
TG	1.01	1.00–1.02	0.05
hs-CRP	1.09	0.95–1.25	0.22
GSP	1.01	1.01–1.02	0.02
PSPW index	0.93	0.90–0.96	<0.01
BI	0.99	0.99–0.99	<0.01

DM, diabetes mellitus; GERD, gastroesophageal reflux disease; TG, triglyceride; hs-CRP, hypersensitive C-reactive protein; GSP, glycated serum protein; PSPW, post-reflux swallow-induced peristaltic wave; BI, baseline impedance; OR, odds ratio; CI, confidence interval. p<0.05.

### Linear relationships between GSP, PSPW, and BI and the risk of developing GERD in DM patients

**Figure 1:**
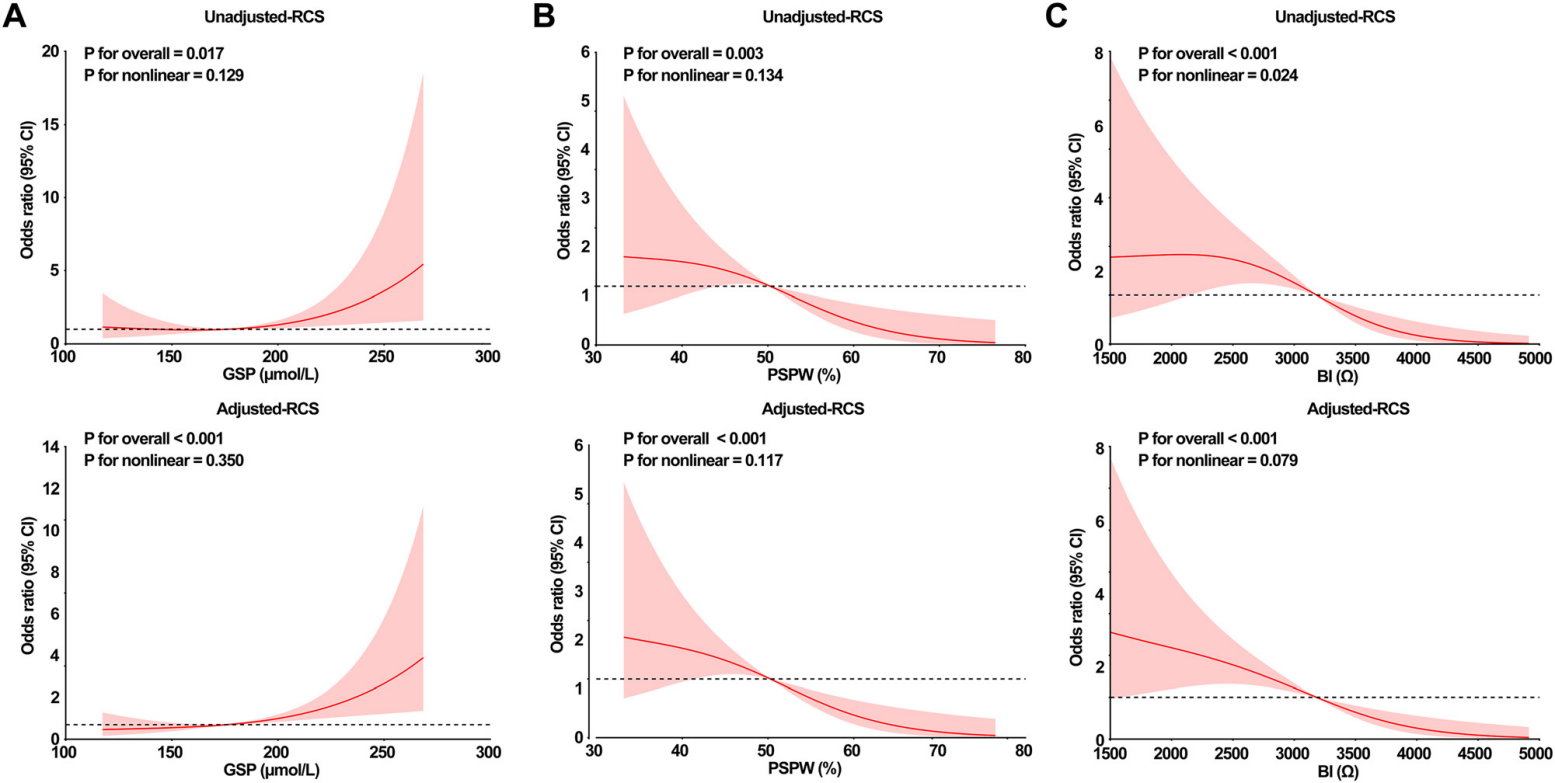
Restricted cubic spline model describing the risk of developing GERD in DM patients based on GSP, PSPW, and BI (p<0.05) A–C, RCS curves for GSP, PSPW, and BI with GERD risk.

A restricted cubic spline model was used to describe the relationship between GSP, PSPW, and BI with the risk of developing GERD in patients with DM. After adjusting for the confounders including age, BMI, smoking and alcohol consumption, DM duration, TG and hs-CRP, it was shown that there was a linear relationship between GSP (p_for nonlinear_=0.599), PSPW (p_for nonlinear_=0.236) and BI (p_for nonlinear_=0.067) and the risk of developing GERD in patients with DM. The risk of developing GERD in DM patients was significantly higher with increasing GSP and decreasing PSPW and BI (Figure 1A–C).

### ROC curve results of GSP, PSPW and BI in predicting the occurrence of GERD in DM patients

**Figure 2:**
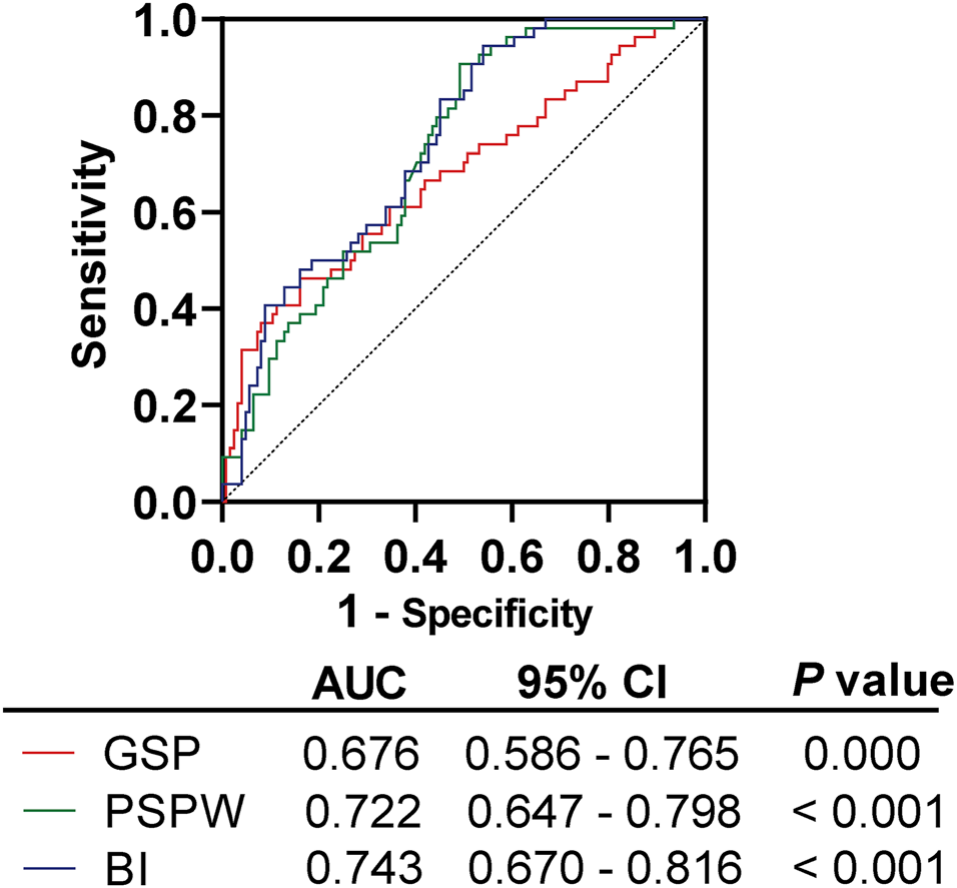
ROC curve analysis of the predictive value of GSP and PSPW for developing GERD in DM patients (p<0.05).

To further investigate the predictive efficacy of GSP, PSPW and BI in predicting the occurrence of GERD in DM patients, ROC curves were plotted with the DM/GERD group as a positive sample and the DM group as a negative sample. PSPW and BI showed good predictive efficacy. The AUC of BI was 0.743 (95 % CI=0.670–0.816, p<0.05), the sensitivity was 94.44 % (95 % CI=0.670–0.816, p<0.05) and the specificity was 45.97 % when the cut-off value was BI <3,653 Ω. The AUC of PSPW was 0.722 (95 % CI=0.647–0.798, p<0.05). When the cut-off value was taken as PSPW <55.23 %, the sensitivity was 90.74 %, and the specificity was 50.81 %. The predictive efficacy of GSP was moderate. the AUC of GSP was 0.676 (95 % CI=0.586–0.765, p<0.05) with a sensitivity of 46.30 % and a specificity of 83.87 % when the cut-off value was taken to be GSP >214.90 μmol/L (Table 4, Figure 2).

**Table 4:** ROC curve results of GSP, PSPW and BI in predicting GERD in DM patients.

Indices	Cut-off	Youden index	Sensitivity, %	Specificity, %	PPV, %	NPV, %
GSP, μmol/L	214.9	0.302	46.3	83.87	55.55	78.2
PSPW index, %	55.23	0.407	90.74	50.81	44.54	92.65
BI, Ω	3,653	0.404	94.44	45.97	43.21	94.99

ROC, curve was used to evaluate the value of GSP, PSPW, and BI, in predicting GERD, in DM, patients. p<0.05. DM, diabetes mellitus; GERD, gastroesophageal reflux disease; ROC, receiver operating characteristic; GSP, glycated serum protein; PSPW, post-reflux swallow-induced peristaltic wave; BI, baseline impedance; PPV, positive predictive value; NPV, negative predictive value.

## Discussion

Gastrointestinal symptoms are frequently seen in patients with DM. Although not usually considered an important cause of death in patients with DM, these symptoms can affect quality of life. Our data showed that the overall prevalence of GERD in diabetic patients was 30.33 %, which is higher than that reported for the general population [19]. GSP, PSPW, and BI were independently associated with the occurrence of GERD in DM patients and all demonstrated good predictive efficacy.

This study confirmed that GSP is an independent risk factor for GERD in DM patients (OR=1.01, p=0.01). Compared to HbA1c, which reflects long-term blood glucose levels over 9–12 weeks, GSP more sensitively detects short-term hyperglycemic states lasting 1–3 weeks [20]. Previous studies have also indicated limitations of HbA1c in DM diagnosis, with GSP or glycated albumin testing recommended as supplementary assessments [21]. Therefore, compared to HbA1c, GSP provides clinicians with more timely information on glycemic fluctuations, potentially serving as a complementary tool for early identification and intervention of GERD risk in DM patients. This short-term persistent hyperglycemia is precisely the core mechanism inducing GERD. GSP levels show significant positive correlations with plasma factor VIIa-antithrombin complex and tissue factor levels, suggesting that elevated GSP may be associated with coagulation dysfunction and activated inflammatory responses [22]. We hypothesize that hyperglycemia accelerates the production and accumulation of advanced glycation end products (AGEs), promotes the release of pro-inflammatory factors, and consequently reduces the mucosal barrier’s resistance to damage [23], 24].

Previous studies have confirmed the association between the PSPW index and GERD. On one hand, the PSPW shows a negative correlation with heartburn symptoms, but no significant association with dysphagia or acid regurgitation [25]. On the other hand, patients with erosive reflux disease (ERD) exhibit lower PSPW than those with non-erosive reflux disease (NERD), while NERD patients show lower PSPW than healthy controls or patients with functional heartburn [13]. This pattern holds true in pediatric patients as well. Children with GERD presenting respiratory symptoms and those with confirmed GERD demonstrate significantly lower PSPW index compared to children without GERD or functional symptoms. Furthermore, the PSPW index shows a negative correlation with esophageal acid exposure time [26], 27], consistent with the findings in this study that the PSPW was lower in the DM/GERD group and that PSPW was an independent protective factor. More importantly, PSPW had excellent performance in predicting the occurrence of GERD and was able to distinguish GERD patients well. It has been found that GERD may be associated with neurological damage. There is a higher chance of esophageal peristalsis inhibition in patients with GERD compared to patients with DM [28], 29]. There may be a link between lower PSPW and increased acid exposure time [30]. PSPW serves as a crucial indicator for assessing esophageal clearance function [31]. Hyperglycemia induces vagus nerve damage, impairing neural signal transmission efficiency and preventing timely initiation of PSPW. By affecting neural and muscular function, hyperglycemia compromises esophageal peristalsis [23]. Additionally, autonomic neuropathy – a common complication of diabetes – may impair neural regulation of the esophagus, resulting in weakened esophageal motility and PSPW dysfunction [32].

PSPW index values and mean nocturnal BI values are positively correlated, as well [33], suggesting a role for chemical clearance in maintaining mucosal integrity. Distal esophageal BI is negatively correlated with dysphagia, but heartburn is not, and other symptoms (such as acid reflux and chest pain) are negatively correlated with proximal esophageal BI [34]. There was a greater consistency in the results of our study. The BI value, as an indicator of mucosal integrity, reflects the degree of integrity of the mucosal epithelial cells and the tightness of intercellular junctions. BI values were lower in patients with DM/GERD than in those with DM. This may be due to the exposure of the esophageal epithelium to reflux in GERD, resulting in tissue damage. Consequently, our hypothesis suggests that the diminished BI values observed in GERD patients stem from acid exposure to the esophageal mucosa. This led us to hypothesize that the reduced BI values observed in GERD patients compared to non-GERD patients resulted from intense acid exposure. Exposure to acid interferes with the complexes of intercellular junctions in the esophageal epithelium. The impaired epithelium exhibits reduced electrical resistance relative to healthy epithelium, leading to diminished MII-pH impedance [35]. This study and several others have confirmed that BI values are lower in GERD patients. However, no previous research has calculated the optimal BI cutoff value for diagnosing GERD. Therefore, we obtained the cutoff value through ROC analysis. The BI cutoff value indicates that when BI is below 3,653 Ω in DM patients, damage to the esophageal mucosal barrier is already present. At this threshold, detection sensitivity is high, making it suitable for early GERD identification. However, specificity is low, necessitating clinical integration with symptoms (e.g., heartburn frequency) or acid exposure time to avoid overdiagnosis. GSP and PSPW cutoff values indicate significantly elevated GERD risk, though sensitivity and specificity remain moderate. Therefore, in practical application, GSP and PSPW can serve as auxiliary indicators for GERD diagnosis. When combined with symptoms and MII-pH monitoring, they enhance diagnostic accuracy. Their utility extends beyond their current value in reflux diagnosis to enable rapid assessment of the likelihood or severity of GERD. Collectively, GSP, PSPW, and BI interact in the pathological progression of GERD in DM patients. Elevated GSP indicates that short-term hyperglycemia, through AGEs accumulation, inflammation activation, and nerve damage, directly reduces BI by impairing the mucosal barrier. Simultaneously, it lowers PSPW by damaging vagus nerve function and smooth muscle function. Reduced PSPW leads to reflux retention, further exacerbating mucosal injury, causing BI to persistently decline, and ultimately triggering GERD symptoms.

Individuals suffering from DM experience a range of complications, with esophageal dysfunction being a frequent occurrence. GERD primarily arises from the temporary relaxation of the lower esophageal sphincter. Excessive weight and obesity independently contribute to the risk of GERD [36]. It has been hypothesized that obesity contributes to GERD symptoms due to increased esophageal sphincter gradient, esophageal hiatal hernia, and intra-abdominal pressure [37]. Hypertension, dyslipidaemia, and poor glycemic control are important factors in patients with DM developing GERD [38]. However, in the present study, there was no significant difference in lipid levels, fasting glucose levels and HbA1c between the DM/GERD and DM groups. There was a statistically significant difference in GSP between the two groups. ROC curves showed that GSP had a better predictive efficacy for GERD. It has been reported that patients with DM combined with neuropathy more often have GERD compared to DM patients without neuropathy, and the prevalence of GERD symptoms is higher in DM patients with neuropathy [12]. As research in this field advances, artificial intelligence (AI) may play an increasingly significant role in improving the diagnosis and management of GERD, offering new perspectives for optimizing MII-pH monitoring. Reports indicate that AI achieves up to 82 % accuracy in identifying PSPW [39]. Applying AI to MII-pH trace analysis for GERD diagnosis holds promise for enhancing the accuracy and efficiency of measuring MNBI and PSPW. Future research could focus on designing studies around MII-pH monitoring data from populations with DM and GERD, developing AI models tailored to this group, and further exploring the potential of combining AI with multiple indicators such as GSP, PSPW, and BI to construct predictive models. This approach could provide new tools for the precise assessment of GERD risk in DM patients.

In this study, some limitations are present. First, this single-center exploratory study featured a small sample size (124 cases in the DM group and 54 cases in the DM/GERD group) with imbalanced group sizes. This not only reduced the statistical power of the tests but also resulted in insufficient stability of the association models between GSP, PSPW, BI, and GERD. Consequently, the cutoff values derived from the ROC curves (e.g., BI <3,653 Ω, PSPW <55.23 %) exhibit limited universality and are difficult to generalize to broader populations. Second, key confounding factors were inadequately controlled. Neither diabetic neuropathy (e.g., autonomic neuropathy, which may directly affect esophageal motility and PSPW function) was assessed, nor were lifestyle factors such as dietary habits, smoking, and alcohol consumption collected. This may have compromised the predictive efficacy evaluation of core indicators. Third, the accuracy of the thresholds is influenced by sample composition. Due to the imbalance in case and control group sizes, the current thresholds serve only as preliminary references and require further validation through large-scale, multicenter studies.

In summary, GSP, PSPW, and BI may be independently associated with the occurrence and progression of GERD in DM patients, and all demonstrate good predictive efficacy. Subsequent research ought to focus on clarifying the fundamental pathophysiological processes of GERD in individuals with DM and their link to the PSPW index and esophageal BI.
